# Opportunities and barriers arising from the COVID-19 pandemic for health campaign integration across immunizations, neglected tropical diseases, insecticide-treated bed nets, and vitamin A supplementation: A qualitative key informant interview study

**DOI:** 10.1371/journal.pgph.0005186

**Published:** 2025-09-29

**Authors:** Alison Krentel, Afzaa Rajabali, Olumide Ogundahunsi, Tuoyo Okorosobo, Eva Bazant, Carol McPhillips-Tangum, Aashka Sethi Sood, Kristin Saarlas, Margaret Gyapong

**Affiliations:** 1 School of Epidemiology and Public Health, University of Ottawa, Canada; 2 Bruyère Health Research Institute, Ottawa, Canada; 3 University of Medical Sciences, Ondo, Nigeria; 4 Health Campaign Effectiveness Coalition, The Task Force for Global Health, Decatur, Georgia, United States of America; 5 CMT Consulting, LLC, Atlanta, Georgia, United States of America; 6 Center for Health Policy and Implementation Research, Institute of Health Research, University of Health and Allied Sciences, Ho, Volta Region, Ghana; University of New South Wales, AUSTRALIA

## Abstract

In low- and middle-income countries, health campaigns play a crucial role in addressing high-priority health concerns such as neglected tropical diseases (NTDs), malaria, vaccine-preventable diseases and nutrition (vitamin A supplementation). Many health campaigns are conducted throughout the calendar year, resulting in multiple campaigns annually in some communities. Campaign integration offers an opportunity to increase efficiencies across programs and limit the strain on healthcare workers, communities, and health systems. The response to the COVID-19 pandemic provided opportunities for campaign integration to mitigate losses from missed campaign deliveries. As the interest in integration grows, there is a need to understand existing barriers, bottlenecks, and opportunities for better integration, whether through co-delivery or increased collaboration. This qualitative study aimed to understand the opportunities and barriers to campaign co-delivery and collaboration from the perspective of 26 stakeholders involved in vaccine-preventable diseases, vitamin A supplementation, NTDs, and malaria programs. Key informants included campaign managers, implementing partners, donors, and decision-makers at the country and state levels in five countries: Côte d’Ivoire, Ethiopia, Guyana, Indonesia, and Nigeria. Results indicated that campaigns were integrated at various levels, from partial integration and co-delivery to fully integrated delivery within existing health services. Most emerging factors were categorized as either facilitating or hindering campaign integration. Enablers to campaign integration included joint planning and appreciation for human resources, while target population variation, prioritization of one intervention by another, and overburdening of healthcare workers, community health workers and community drug distributors were identified as specific barriers. An emerging theme was the importance of leadership, reinforcing the need for country ownership, political will, and positive stakeholder relationships. Further research is warranted to identify optimal combinations of campaign commodities, strategies to ensure stakeholder engagement throughout the integration process, methods to factor in local community context, and the expansion of lessons learned from the COVID-19 pandemic.

## Introduction

In low- and middle-income countries, health campaigns remain an important part of the strategy to address high-priority health areas, including neglected tropical diseases (NTDs), malaria and several vaccine-preventable diseases, and nutrition (vitamin A supplementation). Many of these health campaigns were part of the Millennium Development Goals which outlined global aims to improve child health and reduce malaria [1]. Often funded by international donors and global development partners, health campaigns (also referred to as vertical programmes) can supplement routine health system activities and often rely on the same health workers and community health volunteers to deliver campaigns [2]. Campaigns frequently provide a specific health commodity (treatment, vaccination, bed-net, education/awareness) to communities at a fixed time point with different modes of delivery (house to house, distribution post, specific days) and target populations (age-based, all eligible persons, household level) [3]. The Health Campaign Effectiveness Coalition defines health campaigns as, “Time-bound, intermittent activities that address specific epidemiological challenges, expediently fill delivery gaps, or provide surge coverage for health interventions.” (https://campaigneffectiveness.org/)

Health campaigns provide an opportunity to increase community engagement and train health workers [4]. Through health campaigns, important goals have been reached in the Global Polio Eradication Initiative [5], the Global Programme to Elimination Lymphatic Filariasis (GPELF) [6], trachoma elimination [7] and the reduction of childhood mortality due to vaccine-preventable diseases [8,9]. Despite these positive gains, disease-specific health campaigns may cause duplications, distortions, and interruptions [4] to health systems that are weak *ab initio*, thus impacting the delivery of other health services. Duplications can occur due to the establishment of parallel logistic chains and data management systems. Distortions stemming from higher compensations offered to healthcare workers recruited by vertical programs compared to the general healthcare system can lead to attrition and demotivation among healthcare workers. For instance, the implementation of a Global Fund proposal in Ethiopia resulted in the hiring of local medical personnel through consultancy contracts that provided three times the pay of public sector salaries [10]. This pay disparity leads to the diversion of skilled healthcare workers from the local healthcare system to vertical healthcare programs, ultimately compromising access to general health services and raising equity concerns [11]. Finally, interruptions are caused by diversion of priorities and increased workload due to training sessions and coordination prior to the campaign implementation.

Many health campaigns frequently target the same communities, leading to multiple campaigns throughout the calendar year in some communities. Considering this, campaign integration offers the opportunity to increase efficiencies across programs and limit the strain on healthcare workers, communities, and health systems. Integration can take different forms across the spectrum from partial integration to full campaign integration. Partial integration involves focused collaboration or sharing of specific campaign components between vertical health programs to improve the efficiency and effectiveness of multiple campaigns, but without co-delivery of interventions at the same service delivery point [12]. Collaboration between campaigns can involve various levels of cooperation, such as heightened communication, information exchange, and/or sharing of personnel and resources. An example of this is combining training materials or training days or sharing census information across programs. Full campaign integration involves coordinating most or all typical campaign components (microplanning, registration, logistics, implementation, and evaluation) to allow co-delivery or simultaneous delivery of two or more health interventions at the point of service delivery.

The COVID-19 pandemic revealed weaknesses in our health systems, leading to the reassignment of infrastructure and resources from existing health programs to address the exigencies of the pandemic [13–15]. This redirection caused delays in routine vaccinations, disruptions in bed net distribution, and suspension of mass drug administration for neglected tropical diseases during the early stages of the COVID-19 pandemic [16,17]. Although the focus shifted to COVID-19, the demand for these programs did not diminish. Catch-up campaigns became necessary to limit existing shortfalls in vaccine uptake and address the ongoing needs of these essential health programs [18].

Although integrated campaigns have been occurring for some time, there is a lack of documentation and evaluation of their impact. The barriers, bottlenecks, and opportunities to better integration, whether through co-delivery or increased collaboration, are not yet well understood. These aspects may be typically identified during the course of implementing a health programme, as they are rooted in factors related to the local community, national or regional contexts, as well as the health system.

Before 2020, there was no established platform to identify and promote promising practices among the numerous and varied health campaigns. Recognizing this gap, The Task Force for Global Health (TFGH) initiated the Health Campaign Effectiveness (HCE) Coalition, with support from the Bill & Melinda Gates Foundation, aiming to:

- Foster communication and collaboration among various sectors involved in health campaigns, including country leaders, global stakeholders, donors, and implementing organizations.
- Promote implementation research aimed at identifying, testing, evaluating, and replicating evidence-based practices, delivery models, tools, and approaches relevant and acceptable to country health programs.
- Advocate for policy alignment and collaboration between global partners and countries regarding campaign funding and support, particularly approaches that integrate with broader health systems interventions and primary healthcare.

As the HCE Coalition shaped its implementation research agenda, it became evident that a firm understanding of the potential barriers to campaign integration and the available opportunities would help inform future research efforts at the country level. Our study aimed to assess the effects of COVID-19 on health campaign delivery and examine the opportunities and challenges for campaign co-delivery and collaboration. We gathered insights from campaign managers, implementing partners, donors, and decision-makers at both the country and state/provincial levels across diverse global regions in an effort to answer the following questions:

- What kinds of integration, co-delivery, and collaboration have already been implemented, and what facilitated those actions? How were they received by health workers, national program staff, and the target population?
- Where do informants identify opportunities for integration, collaboration, and co-delivery, and what potential barriers and bottlenecks do they perceive in achieving these objectives?
- What challenges do the informants anticipate in adopting a more formal approach to integration, co-delivery, and collaboration within health campaigns?

## Methods

### Study setting and informants

The present research is an exploratory study using qualitative methods, conducted between October 2020 and January 2021. The recruitment period for this study was from 19-10-2020 to 25-11-2020. Using a purposive sampling strategy, informants were identified in five countries: Côte d’Ivoire, Ethiopia, Guyana, Indonesia, and Nigeria. These countries were selected to represent a range of geographical regions and health systems. Key informants were identified based on their engagement with health campaign integration across five domains - immunization, polio, vitamin A supplementation, neglected tropical diseases, and malaria - and based on their role at the national or state/provincial level of the Ministry of Health (MoH) or within a development or donor agency. The HCE coalition and the research team had existing contacts with individuals working in the selected countries, which facilitated the identification of interview respondents. A matrix was developed to ensure sufficient distribution of informants across the five countries and domains.

### Data collection

An email invitation was sent to potential informants describing the aims of the study and requesting participation. Upon agreeing to participate in the study, informants were invited to an hour-long interview conducted using a cloud-based video conferencing service (Zoom). Verbal consent was given by each respondent before the interview began. All interviews were audio-recorded and conducted in English or French. Audio files were stored on a protected web-based platform. At least two members of the research team were present during each interview to lead the interview and take notes. A topic guide developed by the senior research team (AK, OO, MG, TO) in collaboration with the HCE Program (EB, CMT) addressed the impact of COVID-19 on campaigns; experience with campaign integration; benefits, challenges, opportunities, and risks of integration; decision-making process for campaign integration; role of leadership in the decision-making process; and advice for future campaign integration. Following each interview, researchers discussed the interview, making further notes and observations to be included in the analysis.

### Data processing and analysis

In-depth interviews were transcribed verbatim by three research assistants and entered into NVivo 12 software. The data was coded and analysed for cross-cutting themes. The senior research team and one research assistant worked together on the coding framework and ensured interrater reliability by co-coding a selection of transcripts. When discrepancies arose, the two coders discussed the differences and agreed on a final code. Interrater reliability was high. Results have been anonymized for presentation.

### Ethics approval

The Bruyère Continuing Care Research Ethics Board (REB) in Ottawa, Canada, and the Ondo State University of Medical Sciences Research Ethics Review Committee in Ondo, Nigeria, approved the study. A verbal informed consent was obtained before each interview and the consent form was subsequently emailed to informants upon completion of the interview. Where requested, a transcript was sent to informants.

### Definitions

For this paper, partial integration refers to the collaboration or sharing of specific components between vertical health programs without the co-delivery of interventions at the same service delivery points. Full integration implies coordinating most or all campaign components for co-delivery or simultaneous delivery of two or more health interventions at the point of service delivery [10].

## Results

### Informants’ characteristics

The 26 informants were predominantly male and represented the five health domains with 8 individuals working at the Ministries of Health and the remaining were implementing partners or donors. (Table 1).

**Table 1:** Characteristics of the informants (n = 26).

		Country				
	Characteristics	A	B	C	D	E
Gender	Male (16)	5	2	3	3	3
	Female (10)	0	5	2	2	1
Domains*	Vitamin A supplementation (3)	0	0	1	2	0
	NTD (12)	3	3	1	2	3
	Immunization (9)	2	3	3	1	0
	Polio (6)	1	4	0	1	0
	Malaria (5)	0	3	0	0	2
Affiliations	Donor/Implementing partners (18)	4	4	5	4	1
	Ministry of Health (8)	1	3	0	1	3

*Totals across health domains exceed 26 as some informants worked in more than one domain.

### Impact of Covid-19 on campaigns

Following the World Health Organization (WHO) guidelines at the beginning of the COVID-19 pandemic, all scheduled health campaigns were suspended or delayed [19]. As the pandemic continued, health campaigns were restarted to prevent disease outbreaks, boost immunity, use preventative chemotherapy drugs before their expiration, and mitigate increased disease burden. The challenges informants faced after the resumption of health campaigns during the pandemic included restriction of movement, redirection of human and financial resources, school closures (which precluded school-based health program delivery) and increased financial expenditure. At the same time, informants feared that their operating budgets would be repurposed to respond to the ongoing pandemic. An implementing partner from Country C said, “*We’ve heard that up to 85% of the [national] NTD budget for this year was reprogrammed to support COVID. And they’re [the MoH] expecting a similar amount next year*.”

When asked about the community response to the cessation of health campaigns, informants reported that communities were accustomed to annual campaigns and anticipated them. “*They are now looking forward to receive treatment because they are looking for that one [the treatment]*” said a government official from Country A. An implementing partner from Country A described how the discontinuation of an anticipated program that had been running for 18–19 years impacted the trust between communities and institutions.

In addition, informants reported that some communities expressed concerns about visiting health facilities and coming into close contact with people outside of their communities due to the fear of contracting the SARS-CoV-2 virus. An implementing partner from Country B said, “*there were lots of rumours going around that anyone going to the facility will contract COVID-19, so it affected our immunization programme and other [primary health care] activities.”* In Country D, informants indicated that rumours surrounding COVID-19 vaccine trials resulted in vaccine hesitancy among communities. An implementing partner in country D mentioned that this mistrust “*called into question the whole foundation of immunization*.”

Service delivery disruptions brought by the COVID-19 pandemic resulted in measles and polio outbreaks in some informants’ countries, which led to the resumption of immunization activities in the second half of 2020. The resumption of these activities was an opportunity to consider the integration of different campaigns. A national program manager in Country E surmised that the COVID-19 pandemic elevated campaign integration from an idea or option to a “must” because *“we cannot neglect to deliver health and we realized that there’s a lot of people that can’t reach us.*” Similarly, a program director in Country E stated, “*COVID-19 is really opening up the entire system’s eyes in terms of identifying gaps that need to be addressed.”* Public health surveillance, catch-up immunization, behavior change message integration, coordination, and integration with COVID-19 immunization were identified as opportunities for integration. An implementing partner in Country A said, “*there was a very good logistics working group to make sure that supplies are available for COVID … and then COVID-related health interventions. There was a strong… coordination platform, which we can learn and really scale up or maintain this coordination in the remaining of the year, I mean, I would say couple of years because it’s one of the lessons we have learned*.” Regarding surveillance, an implementing partner in Country C suggested that it would be practical to inquire about whether a child had contracted measles in the last two weeks or a month when asking about COVID-19 cases.

**Fig 1:**
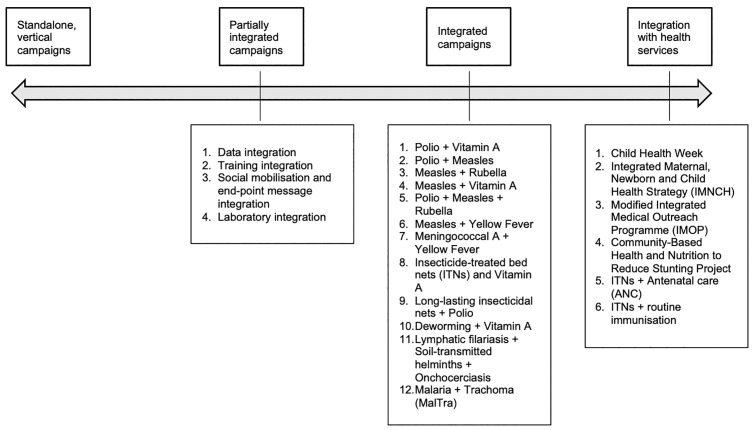
Types of interventions offered in partially and fully integrated campaigns, and campaigns integrated with routine health systems, as described by study informants.

Informants described many pairings of integrated campaigns (Fig 1) ranging from standalone vertical programs to health campaign activities integrated into routine health service delivery within the health system. In decentralised health systems, these routine activities were described more frequently and in greater detail.

### Combinations of interventions delivered in integrated campaigns

Key informants provided descriptions of how campaign integration occurred across five different countries and within five distinct health domains (Table 2).

**Table 2:** Campaign integration described by key informants.

	Vitamin A	Immunization	Polio	NTD	Malaria
**Vitamin A**	n/a	Measles + Vitamin A	Polio + Vitamin A	Deworming + Vitamin A	(Not discussed)
**Immunization**	Vitamin A + Measles	1 Measles + Rubella 2 Measles + Yellow Fever 3 Meningitis A + Yellow Fever	1 Polio + Measles 2 Polio + MR	(Not discussed)	ITN + routine immunization
**Polio**	Polio + Vitamin A	1 Measles + Polio 2 MR + Polio	n/a	(Not discussed)	ITN + Polio
**NTD**	Deworming + Vitamin A	(Not discussed)	(Not discussed)	LF + STH infections + Onchocerciasis	Malaria + Trachoma
**Malaria**	ITN + Vitamin A	ITN + routine immunization	Polio + ITN	(Not discussed)	n/a

Abbreviations: ITN, Insecticide-treated bed nets; LF, Lymphatic Filariasis; MR, Measles-rubella; STH, Soil-transmitted helminth

### Decision-making process for integration

Informants were asked to describe the decision-making process for health campaign integration within their respective countries, including from where the decision to integrate arose. The informants indicated that while it is essential to involve all stakeholders (external and internal) in the discussion on campaign integration, there was a consensus that the central level of the MoH should take the lead on this process.

A national program manager in Country D said, “*Well, I, for one, say leadership belongs to the country*.” An implementing partner from Country D said, “*The only one that has visibility on what everyone is doing is the Ministry of Health. And that’s why the leadership has to come from them*.” Leadership challenges at the central level also emerged from the interviews. An environmental officer in Country B stated that politicians sometimes “hijack” or repurpose programs for their political benefit even when donors fund the majority of the program. One medical officer from Country C highlighted the lack of flexibility within the MoH, stating, “*Our health system colleagues in the Ministry are a bit reluctant to change their set up. So, when we say... ‘can you do services on the weekend Sunday?’ They say: ‘it’s a holiday and we can’t do on a weekend’. And when you ask: ‘can you do on Sunday and take a holiday on Monday?’ Their response is: ‘Oh, that’s not allowed as per the laws, you know*.’”

Donors’ and implementing partners’ contributions to the decision-making process were described as technical and financial support to the government. An implementing partner and former government official from Country C said that the role of “*donors or partners is very important because they can fill the gap, … for example, the gap in the information system or the gap in the advocacy, or in the risk communication*.” However, donor-led decision-making was mentioned as a barrier to integration, “*I don’t appreciate the partner deciding, that he comes with all these orientations...It’s personally those things that bother me*” said a national NTD program manager in Country D.

In Country A, a central-level informant explained the crucial role of stakeholders at the local level: “*The decision-making, in fact, is better done at the local level, where the data makes sense, where the action can be taken immediately, and where the action can be taken by people who know the area, who gather the data,...But most of these, I’m afraid, most of these exist in theory in many places.*”

Acknowledging that the extent of involvement was context-specific, respondents made a distinction for countries with decentralized health systems, as follows: “*In the decentralised setting, districts have quite vast powers. They have good resources also and health is a decentralised subject. So, national government’s role is more of a technical support, providing training guidelines, monitoring, and evaluation. But the implementation and the cost of implementation is to be borne by districts out there. So, districts do have an important voice, I would say, in this whole program. And it works both ways. Sometimes, they say no [to integration].”* (Implementing partner in Country C)

### Factors that need to be considered to ensure effective health campaign integration

Informants highlighted factors that enabled and hindered integration, with some factors serving as both enablers and barriers simultaneously (Table 3).

**Table 3:** Enablers and barriers of health campaign integration.

Theme/Factor	Sub-theme/Specific Factor	Enablers (+) and Barriers (-)
Commodities and Logistics	Availability	+/-
	Issues related to combination of commodities	+/-
	Evidence of integration	+/-
	Timeline	+/-
	Funding	+/-
	Incentives	+/-
Coordination and Communication	Priorities	+/-
	Siloed/vertical program approach to work	–
	Overshadowing (Dominance of one program over another)	–
	Joint planning	+
Contextual issues	Target population	–
	Seasonal patterns of transmission	–
	Setting (urban vs rural)	+/-
Data management	Data collection	+/-
	Indicators	–
Human resources	Appreciation for human resources	+
	Lack of trained HCW/CHW/CDD*	–
	Overburdening	–
	High staff turnover	–
	Volume of information	+/-

*HCW, healthcare worker; CHW, community health worker; CDD, community drug distributor

#### Commodities and Logistics (timeline, funding, incentives).

Alignment of activities, funding, and commodities with each partner’s timeline was identified as a challenge for campaign integration. A malaria program representative at the Ministry of Health in Country E said, “*I might want to integrate with another group or collaborate with another group, but my timings might not be their timings*.” An implementing partner in Country D said, “*Sometimes it is quite difficult to forecast given that some other partners who are involved in providing these commodities in the country also work on an annual basis... It’s difficult to forecast that … when we are applying for a four or five-year period, for instance*.” A national program director in the same country explained, “*When I talk about challenges related to the availability of resources, I link it to the fiscal calendar. We have partners who have the calendar year starting from January 1st to December 30*^*th*^*, other partners who start from April 1 to March 30*^*th*^*, and another partner who starts from September 1st to October 30*^*th*^*... So, our challenge is to match all these calendars*.”

Combining commodities was found to be easier with smaller and easily transportable items and with an efficient supply chain management system. Conversely, bulkier commodities posed an operational challenge. An implementing partner in Country C explained, “**During the polio campaign back in 2018……. in which you have to walk like eight hours in a forest to reach one village for example, bringing the vaccine is already difficult, then bringing the bed nets, yeah... The bed nets are quite heavy. You can…. only bring around five per* *person.”**

Informants identified resource optimisation as one of the major drivers of integration. “*I think that, in order to take a decision about to integrate or not to integrate, to me, it comes down to cost-effectiveness,*” said a donor in Country C. In Country D, an informant stated, “*And in light of scarce resources, the state decided to use more effectively the few resources that were available to us to integrate these NTDs. It is from there that the National Program for NTDs was born*.” However, some concerns were raised by informants about the lack of funds and divergent interests. A polio program coordinator in Country A said, “*As you know sometimes some partners will say: ok, we have only funds for social mobilisation, not for something else.*”

The primary approach for fostering and maintaining motivation in health campaigns typically revolves around the use of incentives for community health workers and volunteers, which may include various forms of compensation such as payments, wages, advancements, or recognition for particular tasks or levels of achievement [20]. Three forms of incentives for community health volunteers, key players in health campaigns, were described during the interviews: monetary, capacity building, and intrinsic motivation. Some of the in-kind contributions included support during post-campaign evaluations, knowledge, and skills transfer. Although the amount varied across countries, informants highlighted that monetary incentives had to be uniform and distributed in a timely manner. One Vitamin A program officer in Country D said, “*When we manage to integrate the [campaigns], we give the same incentive [to community health workers]. That is to say that we do not make an addition, an arithmetic calculation... so, we give the same motivation because the first objective is already to optimize the resources that are not enough*.” In contrast, others preferred non-monetary incentives. A Country A implementing partner stated, “*But this [incentive] is like junk food. You get it now and you need it in the afternoon, you need it tomorrow. But it is not healthy. It doesn’t help the government, it doesn’t help the health worker, it doesn’t help anybody, even the donor and the implementing partner*.”

The lack of evidence around the optimal mix and safety of combined commodities created uncertainties for campaign integration. An NTD program manager (Country D) stated, “*So here we didn’t dare to distribute all the NTD chemoprevention drugs because of the management of the side effects and the difficulty that can occur in attributing a side effect to a molecule*”. Similarly, a medical officer (Country C) said, “*Integrating unknown activities for which you really don’t know what might go wrong is quite risky, you know. So, for example, integrating... lymphatic (filariasis) program with vaccination program, we don’t know who will blame whom for what... How do you investigate and look at these aspects of the program? So, people prefer to play safe. I mean the ministries prefer to play safe and have their vertical [campaigns].”*

However, when there was evidence supporting the safety of combined commodities, the decision-making around integration was reported to be easier. An implementing partner (Country A) explained, “*We also prepared the algorithm for the treatment and so on. So, we did, from the scientific point of view, co-administer both (antimalarial) for falciparum and for vivax, Chloroquine, for example for malaria. And for the trachoma, we are using Azithromycin, a very safe antibiotic… which doesn’t have significant side effects or adverse effects, as such. So, these pharmacokinetic things, that was not really a problem at all*.”

#### Coordination among decision-makers and communication.

Informants voiced concerns about the propensity of organisations to work independently with little collaboration at both central and donor levels. Convening leaders across programs and having them work together was not easy. The director of an immunization program (Country B) said, “*Integrated campaigns require pulling people working on different programs together. Everyone has their own ways of doing business and having to pull them together to provide services in one ten-day period or five-day period is not the easiest task*”. An implementing partner in Country C explained, “*There is not one Director General, there are seven Director Generals in the Ministry. There is one for medical care. There is one for prevention program... and on top of that is the Minister and sometimes it’s not easy to go around to the Minister and ask him to do this coordination work*.”

A lack of alignment of priorities between external partners who tended to focus on one aspect of a program versus the goals of the Ministry of Health was also discussed. There was a perception from some informants that the country goes along with external requests that were not always seen as country needs. A technical advisor working with an NTD program (Country C) said, “*Some of the challenges at least from my experience have been with the donors because a donor usually selects a program or a part of a program that they really want to support. And then all of the funding, all of the program strategy is really focussed on that portion of the program, and it makes it really difficult to integrate.*” The director of a non-governmental organization (NGO) (Country D) explained, “*The difficulty will come when actually you want to do things that the country doesn’t need but that the country accepts anyway. Why? Because behind, there can be funding, per diems. Well, these are some things that are not elements that should drive the final decision of an intervention*.” An advisor at the Ministry of Health (Country A) discussed the importance of considering the communities’ needs and focusing on their livelihoods:

“*Whose agenda is that one? I think that’s most important. Sometimes, it’s national agenda, it’s global agenda. When it goes to me, it might not be my agenda. I have my own priorities of when we are setting priorities on campaigns and so on... For example, when it is harvest time, they [communities] don’t think about any campaign. They don’t like it because their mind is to harvest, to collect their cereals and crops, and so on.”*

“Overshadowing” or the prioritization of one health intervention by another was reported to be a barrier to campaign integration. An implementing partner (Country A) alluded to competing ideals and personal agendas illustrated by those *“trying to take credit for himself or herself*.” One Vitamin A donor (Country C) questioned integration’s impact on outcomes, “*if you are delivering a package, often those review meetings that happen after the campaign has ended, they are not usually looking at all of the coverage of all the interventions to determine what kind of mop-up is needed. There’s usually a primary [intervention] and then the others just sort of follow*.”

Based on their previous integration experiences, informants identified joint consultative planning, review meetings, and open communication as facilitating factors during the integration process.

*“All the partners or stakeholders in the regions, we had the consultative process with them and the roles and the responsibilities of each was clearly spelled out. We had our guideline and with the guideline we had also explicitly outlined the roles and the responsibilities of each and every partner at all levels starting from the federal level to the national level to regional level, zonal level, and district level” - Director of a National Agency (Country A)*

An implementing partner (Country D) explained*, “But when we manage to address these challenges of coordination, to … hold joint meetings for example, we are able to make joint decisions which are based on the same foundations.”*

#### Contextual issues.

Contextual issues included the setting as well as the identification of the target population. Informants were mindful of the seasonal patterns in disease transmission before planning an integrated campaign. An NTD program manager (Country D) explained, “*The season of transmission of bilharzia [schistosomiasis] and filariasis are not the same. These seasons must be taken into account for chemo-prevention to be effective.*”

With regard to the target population, the challenges of conducting integrated campaigns for different age groups surfaced. An NTD senior technical advisor (Country C) said, “*But to try to force coordination or integration when you’re talking about different mechanisms and different groups it’s really complicated.*” A polio stakeholder (Country D) stated, “*We should not integrate when we have different target populations. For example, for immunization, we have the ‘under [age] five’ population and for another intervention, we have other populations. We need to align. If we’ve not the same target population, we should not integrate*.”

#### Data and Data Management.

Data and evidence were found to be crucial in the decision-making process, during and after the campaign, and in the establishment of trust between external and internal forces. An NTD program manager (Country D) spoke on the “*power of* *data which is verifiable… [since it] establishes the trust between your partners and us*.” The Director of Vector-Borne Diseases program (Country E) stated:

“*Because we are looking to merge two things that naturally might not go together or they may very well complement, it needs to be data-driven to really understand how they can mesh properly. To understand and avoid duplication, we need to have data to ensure that we don’t collapse the services. We need to have data to keep track of the goals during implementation and ensure that we are in track to achieving them. We need to be monitoring the data that’s coming in real-time so that we can adjust implementation to really get us back to track if we are tending to slip off track. And so, I really see the role for data is key pre-, during, and even post- implementation in order for us to have a really robust attempt at delivering the quality of the campaigns that we want to deliver*.”

Challenges of data management were described at two levels – at the level where the data is collected and at the donor level where indicators are provided. An implementing partner (Country B) stated, “*… one of the issues that we have in integration is that the forms, the tools are very complex*.” An implementing partner working in the field of immunization (Country D) explained, “*The difficulty is that when each funder comes up with their money to fund a program, they give their indicators.… If there’s not strong leadership at the national level to say: my health worker, these are the indicators that he can track; he can’t track 10,000 indicators…He has his own business. So that’s where integration is crucial*.”

The need to utilise one common census data and coordinate indicators during the planning phase was also highlighted. Drawing on past experiences, an immunization program director (Country B) reported, “*What we realised is the micro plan that are used by the ITN teams and the denominator they used is different from the micro plan and denominator we used in the polio program but if we had planned together, we might not have missed sections or clusters of households and we may not have had as much with refusal. So, for example, the malaria team used the 2006 census data whereas the polio team use that data plus micro plan that we have done more recently to count number of children and our more detailed micro plan that we have done prior to the campaign. So, we were covering larger numbers and they were covering fewer numbers, so we had high numbers of refusal and that affected the implementation*.”

#### Human resources (volume of information, training schedules).

Informants reported that HCWs, CHWs and CDDs appreciated the timesaving benefit and efficiency of integration. A medical officer (Country C) explained, “*At the end of the day, the health workers are very happy if the integration is done because he doesn’t have to do the same walk on the path twice to reach to the same difficult areas and talk to the community.*” The lead of an immunization program (Country B) stated, “*You potentially may not get as much burnout of the field staff, so that they are not running after one campaign or the other.*”

However, high turnover, overburdening, and lack of sufficient numbers of HCW/CHW/CDDs were identified to be barriers to integration. An informant (Country B) discussed attrition within volunteer-run programs as a challenge, “*One of the issues you have to deal with will be to train the 5 or 10% community volunteers that due to attritions would not be there the coming year*.” An NTD program advisor at the Ministry of Health (Country A) stated, “*The next thing you know is that they moved elsewhere, and you may be forced to go to square one.”*

Inaccurate data reporting was mentioned to be a consequence of overburdened HCW/CHW/CDDs. The director of a parastatal agency (Country B) said, “*And when they get tired, they start falsifying figures for you. Because you want to cover 100% polio coverage, right, they will give you the data without actually giving those children those polio drops.*” In the same vein, an implementing partner (Country D) explained, “*And they are not able to provide reliable data because … everyone asks him to do everything and he cannot do everything; a day lasts what it lasts*.”

It was also reported that a high volume of information can lead to confusion and misinformation. An immunization program lead (Country B) said, “*Looking at the capacity of our healthcare workers we know when you give them so much information, they get confused. So, there are issues of capacity gap. When we are having one campaign, they are just trained, you know, do this and it ends there but now you are having integration of three campaigns, the health worker get confused.*” A malaria program manager (Country E) stated, “*If we are trying to put too many messages or too many activities into one then your target population may grasp a part of it or almost sometimes less than half of what you’re trying to get because they are trying to process things that are happening around.*”

### Variation across the health programs

**Fig 2:**
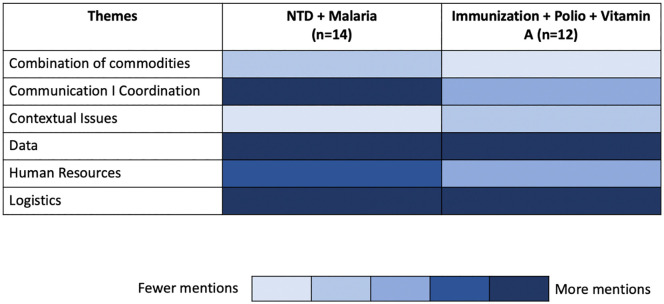
Heat map showing the varied frequency of mentions among study informants across health programs.

To assess how these themes were considered across the five health domains, the subthemes were grouped under their primary theme. Due to the central Ministry of Health grouping many NTD and malaria programs together in these countries, and observing polio, immunizations, and Vitamin A frequently combined, they were also consolidated for reporting purposes. The following heat map (Fig 2) represents the frequency in which the themes were mentioned by informants in these health domains. This should not be interpreted as representing the strength of that issue for informants in that health domain.

There was consistency in the themes raised across countries and health domains. Where there were stronger and more decentralized health systems, more activities were integrated as part of the routine health services. In these cases, for example, child health weeks included not only vaccinations and nutrition but also health information about other conditions, like malaria. Communication and collaboration were seen by all informants as key ingredients for success or as important barriers to integration. The combination of commodities was mentioned more frequently by informants working in NTD/ malaria, likely due to the logistics of combining bed nets with other health commodities, like preventive chemotherapy. Within the NTD domain, there was a concern about combining preventive chemotherapy with vaccines as it would be difficult to tease out any adverse events. Informants working in the immunization domain noted different target populations across campaigns: children versus adults or whole households. When a donor only supported one part of the program or when they had different fiscal years, campaign integration was more challenging.

### Community acceptance of integrated campaigns

#### Appreciation for a package of services.

Informants reported that the community appreciated the package of services offered through integration. One informant (Country E) said, “*They really look out not just for one single team coming in just focussing on one single item, but more expansive and more comprehensive group. So, collaboration in terms of getting to the community it’s one of the most welcoming, I should say, ways to reach out.*” An implementing partner (Country B) explained that lessening the burden of activities helps with acceptance; for example, “*Today we come with an FIPV [Fractional Inactivated Poliovirus Vaccine] activity, tomorrow you are coming with an RI [Routine immunization] intensification. The community is like tired, every time. But now, having integrated, somebody conveniently comes and assesses all these activities and goes back in one visit. You don’t need to come this week, come next week. I think, it also helps the community to really come out and access these [services].*”

Informants also noted that to make the campaigns more appealing, they incorporated commodities such as ITN, consultative services, and integrated messages and health promotion activities. A polio program coordinator (Country D) stated, “*In many countries, I would like to say, we’d like to think of polio fatigue. That’s because we are providing many times polio [vaccines]. People are tired. When you integrate something like Vitamin A, like hygiene items, I think it will be attractive for the population, for the community.*” Speaking about integrated messages, an immunization officer (Country C) said, “*Messages or materials which are produced are all one. You know, they all have common messages that: come to health centres, get your child vaccinated, get your vitamin A drops for your children. So, this is one message which is brought out, you know. It brings synergy. It makes certain efficiencies. It’s attractive to community*.”

#### Increased turnout.

Community acceptance was reflected through the increase in coverage levels. An implementing partner (Country B) said, “*We are really seeing a large turnout. You know yesterday we were discussing at the … and we mentioned the fact that there are communities we had gone to and we saw that immunization was just zero, but with this integration and the available pluses, something as small as a packet of [instant noodle] … it is changing the narrative.*” Drawing on his experience with the lymphatic filariasis campaign, an informant (Country E) stated, “*We had really good coverage; 2017 and 2018 were really very good in terms of coverage. And we had the support. We had some resistance, as always, but we had really good support and good turnout from the schools and from the children, and also from the general population*.”

#### Cement that holds integration together.

The following themes related to leadership, trusting relationships and political will were identified by informants as the “cement that holds integration together” or the “oil that keeps the engine running.”

#### Leadership qualities.

Informants described the importance of leadership in driving the decision to integrate. Informants attributed the following qualities (S1 Table) to a good leader: availability, accountability, advocacy, delegation, flexibility, humility, knowledge, open-mindedness, peer recognition, organisation.

*“We know that leadership is key whether you are trying to eradicate polio or whether you are trying to integrate programs to work. If the leaders don’t appear coordinated and if they are not working from the same work plan, then it does not flow down to the staff, and it does not work.”* Immunization program director (Country B)

*“If people don’t have good leadership, the institutions will be an empty furnace in spite of all the literature and pilots that are in the pipeline... And so human relations are more important because that’s what builds trust.”* Ministry of Health official (Country D)

The quality of an institution was also discussed as a factor of successful integration. An implementing partner (Country D) said, “*I don’t think one person can have visibility on all aspects of managing a program. A program is managed in a way. It’s managed in a financial way, it’s managed in a technical way, it’s managed in terms of human resources. So, I think that it’s not for me a person but it’s a structure. A structure that can be a ministerial structure, therefore dependent on the Ministry of Health, which is able to know one-partner mapping. Who works where? Who does what? With what funding? So that I think that in many countries, in fact, we don’t have this cartography.*” An informant at the Ministry of Health (country A) talked about sustainability, “*Champions, my view is at the beginning yes. We need them to come up with proof of concept, to help us win minds and hearts of other key stakeholders, and so on. But in the long term, I prefer to work on the systems.*”

#### Political will.

Informants recognised that it is essential that leaders at the national level take ownership and support the decision to integrate campaigns. An implementing partner (Country A) stated, “*You can expect the Minister or the State Minister to show up on the TV and declare to everybody that this week is dedicated to MDA. So, this means a lot in terms of advocacy, in terms of awareness creation, in terms of enhancing political commitment at various levels including the highest level of political leaders and so on.*” A donor (Country C) said, “*Commitment to high, you know, coverage or commitment to the interventions to deliver is incredibly important as an enabling environment*.”

#### Positive relationships.

Informants observed that having positive relationships with partners facilitated the decision-making process.

*“I like to say that beyond institutions, it is human relationships that facilitate activities. Institutions can exist, but if the people who animate them do not have a good understanding of the thing and they do not have a good collaboration, it is certain that in the execution of things, there will be difficulties and blockages because I always say the problems of good ideas are easier to solve than the problem of people. But for questions of sustainability, if not at our level, the coordinators just have to decide to get together to do it. And we have a good relationship with the EPI [Expanded Programme for Immunization] coordinators. A very good relationship. It’s phone calls from me and then things move forward. So, we also have a very good relationship with the national nutrition program.”* NTD program manager (Country D)

*“There was no conflict of interest. There was no competition among partners, but there was a sense of complementarity. We were trying to use the skills and the resources and expertise of each of the partner into this mix. It’s the kind of synergy, you know, synergetic. We were trying to pull everything together and deliver it effectively and efficiently to the communities that are requiring it.”* An implementing partner (Country A)

### Advice to colleagues considering integration of health campaigns

At the end of the interview, informants were asked what advice they would give to colleagues in another country who were considering integrating health campaigns (S2 Table). Advice covered specific activities such as microplanning, risk assessment, timelines, and guidelines. Advice is also related to communication, ownership and ‘thinking outside the box.’ One implementing partner (Country A) cautioned against integration for integration’s sake, *“We should identify which things can be integrated and which things cannot be integrated. Everything cannot be integrated, and it doesn’t work that way. And that will alleviate anxiety and will help to maximise the benefits.”* A national program manager (Country D) warned, *“It should not be a fad... integration peaked in 2014, where we were only talking about integration, integration, integration. But there are many aspects that had not been taken into account.”*

## Discussion

Health campaigns faced a range of challenges due to the COVID-19 pandemic. Campaign planning and operation were affected by factors including movement restrictions, reallocation of resources to COVID-19 activities, school closures, heightened expenditures on COVID-19 mitigation measures, and community skepticism towards visiting healthcare centers or utilizing services. NTD programs were amongst the most severely affected programs, threatening the gains achieved in the previous years [14,21]. In May 2020, WHO, Gavi and UNICEF estimated that at least 80 million children were affected due to disruptions in routine immunizations [16]. Two-thirds of additional malaria cases in 2020 have also been attributed to COVID-19 disruptions [22]. Informants mentioned various campaign integration opportunities that arose during the pandemic, including disease surveillance, catch-up immunization, behavior change message integration, and coordination. For some, this type of integration was a novel way of working and reaching otherwise hard-to-reach communities and reversing some of the losses resulting from the pandemic.

Our study revealed that campaign integration occurred at three levels: partial, co-delivery, and full integration with health services, using various approaches and combinations. In some cases, integration was already happening at the lower level of the health system, yet there was insufficient information available to assess how this is done. Most factors emerging from the thematic analysis could be interpreted as both facilitating and hindering campaign integration. Campaign integration was facilitated by enablers such as collaborative planning and recognition of human resources. However, differences in target populations, prioritization of one intervention by another, and the overburdening of HCW/CHW/CDDs were identified as specific barriers.

As such, not all integration is the same, so contextual interpretation is essential. Effective elements of campaign integration are reliant on specific interventions, and local, national, and global factors [23]. Examples of intervention-specific contexts include varying target age ranges, seasons, methods of dissemination, delivery modes, and durations. Local/district/national baseline acceptance, epidemiology, capacity, remuneration strategies, financing, and decision-making policy may also differ. Lastly, global operational guidance, dialogue, incentives, awareness, and priorities may also be at fundamental odds. Identifying context-specific overlap and opportunities for integration, mitigating discordance between donor programs and intended recipients, assuring linkage to national and global policy, and addressing competing ideals must be done through a people-centred approach [24,25]. People-centred integration must be the foremost prerequisite from which all effectiveness can be judged and can serve as a potential unifier and nidus to create an enabling environment to overcome potential barriers [24].

Leadership emerged as a crucial theme in all interviews. In the literature, leadership is regarded as an essential component of the health system [26–28]. Leadership and governance are considered one of the six building blocks in the WHO health system framework [26]. The role of leadership in integration is notable as the study revealed that integration appeared to be more person- and relationship-driven than policy-driven. Because the sustainability of integrated campaigns depends heavily on relationships between partners rather than institutions, such integrations remain fragile.

Informants emphasized the need for country ownership, political will, and positive relationships between stakeholders. Chunharas and Davies propose the idea of interactive leadership between various actors within the health system, including communities and civil society [29]. In our study, we discovered that various entities were influential in driving the decision to integrate. These included the Ministry of Health, professional associations, and civil society groups within the country, as well as external actors such as development partners and donors. However, many informants noted that the process of campaign integration should be led by the central level.

There is some urgency to understand the mechanisms of effective campaign integration. As many programs reach the endgame (some NTD programs, polio campaigns, malaria elimination), integration will be needed to increase efficiencies in finding and reaching those individuals left behind by public health campaigns (the zero dose child in immunization and the never treated in mass drug administration programs for NTDs). In addition, integration offers an attractive platform for combined disease surveillance after campaigns have stopped. Finally, global documents like the WHO NTD 2030 Roadmap highlight the importance of integration and country ownership of programs as we approach the Sustainable Development Goals deadline, yet there are few documented examples of how to effectively institute and maintain integration [6]. Continued research and documentation of real-life cases will aid in moving the integration agenda forward.

This study revealed the opportunities COVID-19 brought to integrate campaigns and factors that are important to consider when planning an integration. Drawing from our findings, we provide recommendations for Ministries of Health, development partners, and donors (Box 1). Additionally, we propose the following implementation research questions for future studies:

- Is there an optimal combination of commodities that can be integrated?
- How do we engage all stakeholders, including communities and HCW/CHW/CDDs, throughout the entire process of integration?
- What lessons can be learned from unplanned and undocumented partial or full integration taking place at the field level? How can these experiences be documented and shared?
- How can we best factor local context and seasonality into the timing of integrated campaigns for optimum outcomes?
- What is the unique set of training competencies across all health workers/volunteers?
- How can we sustain/expand/learn from campaign integration innovations that emerged during the COVID-19 pandemic or other public health crises?
- How do we ensure integration is sustained, when appropriate? How can integration be supported by funding agencies? How can integration be included in national strategic and operational plans?

**Box 1. Recommendations for Ministries of Health, partners and donors**

**Table:** 

Ministry of Health	Development partners and donors
Assess ongoing integration, including successes, failures, and learnings. Review areas where integration is needed to boost participation and coverage Commission implementation research on the barriers and bottlenecks to health campaign integration. Consider ways to strengthen leadership and training for Ministry of Health management. Foster regular communication across sectors, health campaigns and partners/donors to facilitate collaboration. Harness communities’ and community-based providers’ experiences and preferences for campaign integration	Gather evidence on successful health campaign integration from other countries and share it with the Ministry of Health. Ensure that ownership of integration lies with the Ministry of Health and is part of a sustainable approach towards health care delivery. Consider novel ways of coordinating across partners and donors to ensure efficiencies in data management and harmonization of incentive structures for health providers and volunteers. Provide opportunities for open communication across Ministry of Health campaign managers and other partners/donors to facilitate the negotiation process.

### Limitations

Due to the pandemic, no community-level data collection was possible. As a result, the community perception of campaign integration was conferred only by national and district-level personnel. While efforts were made to ensure the inclusion of a comprehensive range of health domains in each country, achieving this goal was not always feasible.

## Conclusion

Out of necessity, the COVID-19 pandemic provided opportunities to integrate health campaigns across multiple disease priorities. These varied integrated health campaigns encompassed immunizations, polio, malaria, vitamin A, and neglected tropical diseases. Integration involved collaboration, co-delivery of interventions, and varying degrees of integration with routine health services. Leadership qualities and other factors both enabled and hindered the integration of health campaigns. Moving forward, Ministries of Health and partners should carefully consider how interventions can be delivered efficiently and effectively together, taking into account the enablers and barriers highlighted in this study.

## Supporting information

S1 Table Leadership qualities needed for integration. (DOCX)

S2 Table Advice of Study Informants to colleagues in another country considering integrating health campaigns. (DOCX)

S1 Checklist Inclusivity Checklist. (PDF)
