# Supplementary material for: Opportunities and barriers arising from the COVID-19 pandemic for health campaign integration across immunizations, neglected tropical diseases, insecticide-treated bed nets, and vitamin A supplementation: A qualitative key informant interview study
Source: PLOS Glob Public Health. 2025 Sep 29;5(9):e0005186. doi: 10.1371/journal.pgph.0005186 (PMC12478929; doi:10.1371/journal.pgph.0005186)
Supplement: S1 Table — (DOCX) [file pgph.0005186.s001.docx]

**S1 Table. Leadership qualities needed for integration**

| **Qualities** | **Quotes** |
| --- | --- |
| Availability | “He or she must have... availability.” |
| Accountability | “And accountability. This is also another area where... when you perform a thing, you have to be accountable for that as well.” |
| Advocacy | “We had these high-profile people coming to that launching meeting, that meeting being transmitted by radio, television, and so on. So, these kinds of mobilisations, sensitisations, and consultations, and involvement of high-profile people in the program made it easier for us to get acceptance by the community.” |
| Delegation | “I am a program coordinator. I know when it comes to data management, I'm not competent for that. There's someone who is competent for all of that and for us to be efficient. And so, my role is to ensure that the activity is carried out according to the rules.” |
| Flexibility | “Here local leadership, I’ve seen, makes a difference. We have seen activities done when convinced to local authorities: done on Sundays, done on Church days, activities conducted from six to eight or done in malls and done in major shopping areas, in recreation parks.” |
| Humility | “For this kind of integration, where everyone sometimes thinks that he must have the monopoly of an intervention, the first thing first of all is humility. You have to be humble. You have to be humble and you have to know that by integrating you always learn from others.” |
| Knowledge | “I think that person really needs to be focused, he really needs to believe that integration works so that when you are having those people that they don’t bring constructive criticism, you try to just overlook them and forge ahead. I think, you need to have that leader who is focused and ready to go ahead and face the risk and address them as they come because there will always be challenges on the way and you need to just be able to say, I am ready for it come up with some mitigation plans, if this happens, this is the way I will do it” |
| Open-mindedness | “One of the major skills that are needed is the ability to pull, bring people together and let them see what the vision is, If you cannot bring your people together and share the vision and let them catch on and be passionate about the vision, then forget it, they are not going to be any followers. So, it’s the ability to listen to what the experts are saying, speaking with one voice with your expert who advised and then ensuring that the vision is well laid out without confusion” |
| Organisation | “Initially the first phase did not work out so well, but when the leaders came together and everybody said we have to do this together, let have one checklist both for the polio program and the IMOP, let’s have one list of ten (10) things that everyone must do... this really made a big difference” |
| Peer recognition | “You should be very transparent. You should be very accountable, and you should not take credits of anybody else. So, if you’re that kind of person, the leadership quality really matters very much.” |
