# Supplementary material for: Opportunities and barriers arising from the COVID-19 pandemic for health campaign integration across immunizations, neglected tropical diseases, insecticide-treated bed nets, and vitamin A supplementation: A qualitative key informant interview study
Source: PLOS Glob Public Health. 2025 Sep 29;5(9):e0005186. doi: 10.1371/journal.pgph.0005186 (PMC12478929; doi:10.1371/journal.pgph.0005186)
Supplement: S2 Table — (DOCX) [file pgph.0005186.s002.docx]

**S2 Table. Advice of Study Participants to colleagues in another country considering integrating health campaigns**

| **No.** | **Advice** | **Comments** | **Quotes** |
| --- | --- | --- | --- |
|  | Collaboration | Across various levels | “But rather than direction, if it is consultative, people will buy-in into the process and then you start with something more. What if we do the planning together? What if we exchange experience together? What if we look for things which can be integrated? Then when people are asked to contribute, everybody will come with open hearts, with open hands. And then identify things which maximise efficiency. If am going to spend only 50% of my resource and get the thing done, I am pretty sure that nobody will say no to that type of partnership, to that type of engagement. So, look for things which can really win minds and hearts of everybody around the table.” |
|  |  | Community engagement | “The thing is we have to go down. We should take the pain of going down all the way to the village and studying the way they lead their day-to-day life and maintain it as it is.” |
|  | Guidelines and evidence | Need for scientific evidence of integration and cost-effectiveness studies | “I think it’s good if you can have examples that are good practices. As I mentioned before, sometimes people don’t even know that they are integrating, but if you were to really assess it, they have integration at different levels” |
|  | Microplanning | General advice | “All way I would say is give time for planning, planning, planning and by that I mean the logistics and the micro plan, ensuring that all the teams, when I say the teams, the programs that are involved are pulled in from the beginning and they have one plan together... give enough time to plan, bring all the various programs or the disease or the whatever activities you are there integrating together” |
|  |  | Clear goals and objectives | “I think the first thing that I’ll say to them would be that one, you should understand your program and what you are hoping to achieve because it’s very easy when you are having collaboration to lose sight of what your goals are and get sidelined or sidetracked from your goals.” |
|  |  | Mapping out actors | “What would have, you know, helped us would have been, you know, really mapping out the actors in the delivery of Vitamin A and deworming.” |
|  |  | Funds | “And really at the end of it, the effectiveness of a program like this also depends on good support in terms of the financial support on a regular basis.” |
|  |  | Human resources | “And then, of course, the issue of the human resource - knowing ahead of time. I would say we really have to weigh the planning and really be sure this can be implemented because nationally we just sit there and say everybody, we want you to implement in one phase, without the knowledge of whether you are going to have enough healthcare workers or not. But I think three months from now I want to implement such an activity, I really need to know the health worker, the human resource capacity across the state ahead of time to really see whether the state can do it at once or they need to phase, and I will give them timelines on how to phase.” |
|  |  | Logistics | “If you look at supplies, they are very sensitive, heavy and also to distribute to hard-to-reach areas. So, this logistics plans should be in place. If it is not properly planned, then the quality of the campaign would be compromised.” |
|  |  | Surveillance | “we just have to watch out for what are the things that might go missing when you do integration... we know that different NTDs are at different stages of elimination and control but the surveillance systems later on are not as well designed and developed. So, not to sort of not to lose the gains that have already been made for years and years. But when we come to the, you know, sort of the last stages of controlling or eliminating or eradicating some diseases, we do not develop robust surveillance systems and that’s where we can see that we will not be able to sustain the gains.” |
|  |  | Timeline | “Before starting an integrated campaign, I think it's to have the right dates, because if it's different directions you have to already have the dates of the different campaigns and then discuss those dates.” |
|  | Open communication |  | “We shouldn’t put things as if everything is rosy. It’s not going to be rosy. So, from the outset, we have to communicate openly that these are the merits, and these are the demerits. And the merits outweigh because of this and that... the first of couple of years are going to be tough but in due course the return on investment is immeasurable.”” |
|  | Phased approach |  | “I think uh it is better to start small because once we start the journey, this is like a long trip together. You sit with somebody, next to somebody. If you start with good greeting, you are likely to enjoy the flight. But if you are not willing to just ask somebody to the next seat, then you are unlikely to enjoy the flight. So, my advice would be start small. Start with things which are acceptable for everyone. It could be very minimum. It could be - let us meet once a month.” |
|  | Risk assessment |  | “I think that persons need to go through different scenarios, assess the different risks involved, and to be sure that benefits outweigh those risks.” |
|  | Taking ownership |  | “From the management, it’s the leadership and the ownership of the national counterpart. I’ll just stick to polio, that people think in many country polio is a WHO and UNICEF business. In countries, I think that to get the ownership and the leadership from the national counterpart is very very important for the success.” |
|  | Targeted approach |  | “You know the last four last years and this year I assessed vaccine hesitancy in [six African countries]. I did a vaccine hesitancy assessment, and you can't imagine sometimes what comes out and can be fixed but easily. But in fact, we want to have the miracle solution to solve everything. I say no. The populations are different. So, we're going to need interventions that are different.” |
|  | Thinking outside the box |  | “So, again as I said earlier, let’s think out of our box and learn to work with others, with partners. There are a lot of lessons to learn and also from elsewhere. So, bring those ones and contextualise that one to the local level. So, for me, it’s just to be open-minded and address health problem in the community.” |
|  | General Advice |  | “It should not be a fad... integration peaked in 2014, where we were only talking about integration, integration, integration. But there are many aspects that had not been taken into account.” |
|  |  |  | “And lastly, we should identify which things can be integrated and which things cannot be integrated. Everything cannot be integrated, and it doesn’t work that way. And that will alleviate anxiety and will help to maximise the benefits.” |
